# Anticipating the third century of the National Library of Medicine: a call for engagement

**DOI:** 10.5195/jmla.2017.190

**Published:** 2017-04

**Authors:** Patricia Flatley Brennan

In nineteen years, the National Library of Medicine (NLM) begins its third century—that means that the master’s of library and information science (MLIS) graduate student who will be entering study in 2036 is three years old about now. What opportunities await our emerging librarian? What is the foundation of the NLM of 2036 that will ensure that it continues to serve medicine, health care, discovery, and citizens? Finally, what guidance can librarians and information professionals offer to ensure that the foundation is well grounded, robust, nimble, and enduring?

As the fourth appointed director of the modern NLM, I seek your wisdom and insights as we launch a strategic planning process in anticipation of our third century. The health sciences library community represents a critical partner on this journey. Here is my hope for how you can help us:

1. Guide us in developing resources to create collections, improve discovery, and accelerate dissemination
2. Help us envision a future where information serves as a platform for discovery and an enabler of action
3. Join with us to design acquisition and dissemination strategies that ensure the availability of information at the point of need
4. Partner with us to create the public policies that promote human and machine access to better scientific information and health data
5. Keep us true to the core library values of servicing the public in the places and ways that are most useful to them

Librarians are critical to all aspects of the mission of the NLM. Many in the health sciences library field are familiar with our most widely used services like PubMed and MedlinePlus and with the National Network of Libraries of Medicine. Some are also familiar with other aspects of the NLM that need more awareness of and attention from librarians and other health information specialists. We have two very large intramural research enterprises: the Lister Hill National Center for Biomedical Communications (LHC) and the National Center for Biotechnology Information (NCBI). LHC, established by a joint resolution of Congress in 1968, conducts research in clinical data standards and electronic medical records, advanced imaging, knowledge representation, and new ways to enhance search through integrating images and natural language processing. NCBI, created through federal legislation in 1988, has as its mission to develop new information technologies to understand the fundamental molecular and genetic processes that control disease. Ontologies, curation, search, and retrieval activities are all parts of their endeavors. We also run a large extramural grant program that supports basic research and training in biomedical informatics, funds the addition of informatics professionals to other National Institutes of Health–funded research programs, and provides the funding for the regional medical libraries (RMLs). Our outreach programs meet people and their information needs from refugee camps to Native American pow wows to intercity churches. There are places for librarians everywhere.

Now is the time for health sciences librarians to partner with our informatics and basic science colleagues to set forth the future of the NLM, launching new initiatives well grounded in a sound history. Data and data science challenges—including acquisition, storage, dissemination, and protection—must be faced with systematic strategies that ensure discoverability while protecting the integrity of the data. New stakeholders, from children to those with intellectual disabilities or low literacy skills, need our attention. We need your talent, your discernment, and your efforts.

Over the next year, I will be attending several regional and national meetings of medical librarians and hope to hear from as many of you as possible. I will be dialing into RML webinars and calling into the network working group meetings. I will also be visiting most of our university-based training programs, and that, too, may offer an opportunity for a conversation. You can always reach me at patti.brennan@nih.gov or follow me on Twitter (@NLMDirector). My blog, *Musings from the Mezzanine,* was launched beginning of this year. Perhaps you will use that opportunity to write a guest blog informing me and the world of the future NLM that you anticipate!

